# Pangenomic and machine learning approaches to divergent pathogenic strategies in avian *Mycoplasma* species: evidence and limitations

**DOI:** 10.3389/fmicb.2026.1936311

**Published:** 2026-09-30

**Authors:** Miao Yin, Qingrong Jiang, Xing Yu, Qian Wu, Yu Xia, Xin Zhao, Miaoxi Deng, Xuemei Ding, Jinglong Yang, Xiwen Chen, Xuefeng Qi

**Affiliations:** 1 Faculty of Agriculture, Mianyang Teachers’ College, Mianyang, Sichuan, China; 2 Department of Veterinary Medicine, Northwest A&F University, Yangling, Shaanxi, China; 3 Engineering Research Center for Monitoring and Control of Major Swine Diseases, Sichuan Provincial Department of Education, Mianyang, Sichuan, China; 4 Sichuan Sundaily Ecological Food Co., Ltd., Mianyang, Sichuan, China; 5 Institute of Animal Nutrition, Sichuan Agricultural University, Chengdu, Sichuan, China; 6 Chongqing Academy of Animal Sciences, Chongqing, China

**Keywords:** antimicrobial resistance, avian mycoplasmosis, host-pathogen interactions, machine learning, *Mycoplasma gallinarum*, *Mycoplasma gallisepticum*, *Mycoplasma synoviae*, pangenomics

## Abstract

Avian mycoplasmosis, mainly caused by *Mycoplasma gallisepticum* (MG), *Mycoplasma synoviae* (MS), and *Mycoplasma gallinarum* (MGn), remains a persistent constraint on poultry production. These species co-circulate in flocks but differ in tissue tropism, host responses, and clinical outcomes. This review examines how pangenomic analysis and machine learning (ML)-based feature prioritization can clarify these differences while separating computational associations from experimentally supported mechanisms. We first summarize genome reduction, structural variation, accessory gene content, and antimicrobial resistance architecture, and then evaluate ML applications in classification, feature selection, network inference, and candidate prioritization. The evidence is strongest for MG, which combines respiratory adhesion, TLR2/MyD88/NF-κB-mediated inflammation, NLRP3 activation, and oxidative injury. MS appears more synovium-adapted, with evidence of fibroblast reprogramming, ER stress-related responses, and interference with antiviral signaling. MGn remains insufficiently characterized because complete reference genomes, standardized infection models, and functional validation data are lacking. We conclude that pangenomics and machine learning are useful only when linked to validation: They can define candidate repertoires and rank testable hypotheses, but they do not establish pathogenic mechanisms. A controlled three-species comparison and a complete, well-annotated MGn reference genome are the most immediate priorities for addressing the gaps that currently prevent confident cross-species comparison.

## 1 Introduction

Avian mycoplasmosis remains an economically significant constraint on poultry production worldwide, with losses attributable to reduced growth performance, decreased egg production, condemnations at slaughter, and recurring antimicrobial treatment costs (Mugunthan et al., 2023). In total, three species account for most of the disease burden in commercial flocks: *Mycoplasma gallisepticum* (MG), *Mycoplasma synoviae (MS)*, and *Mycoplasma gallinarum (MGn)*. These pathogens share a host and, in many production environments, co-circulate in the same flocks, yet they differ in the tissues they colonize, the host responses they elicit, and the disease syndromes they produce. MG colonizes the respiratory epithelium through phase-variable adhesins, including GapA, CrmA, and members of the VlhA lipoprotein family, and inflammatory pathology is driven largely by TLR2/MyD88/NF-κB signaling (Wang et al., 2023). MS is more strongly associated with synovial and systemic disease; the results indicated that apoptosis-related, inflammatory, and matrix remodeling pathways were markedly altered, with dihydrolipoamide acetyltransferase (E2) identified as a virulence-associated factor (Liu et al., 2020). MGn is regularly detected in commercial flocks, but its pathogenic mechanisms remain poorly characterized (Leigh and Evans, 2026). Throughout, we retain the conventional species names (*Mycoplasma gallisepticum*, *M. synoviae*, and *M. gallinarum*) for consistency with the primary literature. Under the current NCBI taxonomy, MG is assigned to *Mycoplasmoides*, whereas MS and MGn are assigned to *Mycoplasmopsis.*

These divergences are notable precisely because *mycoplasmas* operate within severely reduced genomes. Across the genus, genome sizes range from approximately 0.58 to 1.35 Mb, with corresponding losses in biosynthetic, DNA repair, and regulatory capacity (Razin et al., 1998). MG, MS, and MGn therefore provide a useful comparative system: Closely related, obligately host-adapted organisms with limited coding capacity, yet distinct tissue preferences, interaction patterns, and clinical outcomes. This comparison also addresses a broader microbiological question: How host-adapted bacteria with reduced genomes generate distinct tissue tropisms and disease outcomes.

Prior reviews have addressed MG pathogenesis (Zhou et al., 2024; Kachabi et al., 2025), MS-associated synovial disease (Liu et al., 2020; Rufai et al., 2025), and MGn genomics largely as separate topics. This separation partly reflects the methodological constraints of single-pathogen experimental models and static gene inventories. These approaches are useful but poorly suited to explain how gene content variation, structural changes, and host-specific regulatory responses combine to shape divergent phenotypes (Du et al., 2025; Wang et al., 2021).

Pangenomic and machine learning (ML) approaches have begun to address this gap, but neither is yet mature enough in avian *mycoplasma* research to support strong mechanistic conclusions. Pangenomics can map species-specific gene repertoires, identify structural variants, and trace evolutionary trajectories across strains without requiring alignment to a single reference (Domingo-Sananes and McInerney, 2021). Complete genome sequencing of MS (strain 53) revealed a 799-kb chromosome carrying an expanded vlhA hemagglutinin gene family—a species-defining surface-antigen repertoire that single-gene approaches could not have captured (Vasconcelos et al., 2005). ML methods complement this by prioritizing which features in high-dimensional genomic or transcriptomic datasets associate with phenotypes of interest, as demonstrated in antimicrobial resistance (AMR) prediction and candidate virulence-gene identification across bacterial pathogens (Kim et al., 2022; Do et al., 2024). The interpretation of both approaches, however, remains conditional on data quality, sample size, and experimental follow-up. These constraints are especially limiting in a field that still lacks complete reference genomes and controlled, multi-species infection experiments (Do et al., 2024; Domingo-Sananes and McInerney, 2021).

This review has four objectives. It characterizes the pangenomic landscape of MG, MS, and MGn, with emphasis on genome reduction, structural variation, horizontal gene transfer (HGT), and antimicrobial resistance architecture. It then evaluates ML approaches used in *Mycoplasma* research as problem-driven strategies rather than as an algorithm survey, focusing on applicable tasks, data requirements, interpretability, and current limitations. Against this methodological background, it compares evidence for species-specific pathogenic mechanisms across four dimensions: Metabolic reprogramming, adhesion and invasion, immune evasion, and host stress responses. Finally, it synthesizes these findings into a comparative framework, identifies where evidence supports mechanistic inference and where it does not, and specifies the experimental and infrastructural investments most needed to advance the field.

Species selection followed three criteria: Relevance to commercial chicken production, occurrence in the same host, and established or suspected involvement in the major respiratory and synovial *mycoplasma* infections of chickens as primary members of the disease complex. MG and MS meet all three criteria (Kleven, 2008). *M. meleagridis* and *M. iowae* are primarily turkey-associated (Kleven, 1998), while *M. gallinaceum*, although chicken-associated, showed no primary pathogenicity in specific-pathogen-free chickens, with its effects being limited to exacerbating coinfection with infectious bronchitis virus (Adeyemi et al., 2018). MGn is retained as a low-evidence comparator. It is repeatedly isolated from commercial chicken flocks and associated with respiratory disease in coinfection settings (Wan et al., 2010; Leigh and Evans, 2026). Its first complete reference genome, however, was reported only recently (Leigh and Evans, 2026), so its mechanisms are currently understood primarily through comparative genomics and machine learning approaches. MGn is therefore retained not as a third data-rich species but as the case that most directly tests the review’s central premise because computational prioritization earns its value exactly where bench evidence is thinnest.

## 2 Genomic landscape of avian *mycoplasmas*

### 2.1 Genome reduction and pangenomic dynamics

*Mycoplasmas* occupy an extreme of the prokaryotic size range. With genomes of 0.58–1.35 Mb, they have lost most biosynthetic and DNA repair pathways, along with the majority of transcriptional regulators found in free-living relatives (Razin et al., 1998; Papazisi et al., 2003). Whether genome reduction in obligate parasites primarily reflects adaptive selection for streamlining or mutational drift under relaxed purifying selection remains a subject of debate (Razin et al., 1998). These pathogens depend on their hosts for metabolites they can no longer synthesize, tightly coupling pathogen fitness to host resource availability. Compromised DNA repair concentrates mutation at surface-exposed lipoprotein loci, where immune pressure generates the strongest selective gradient (Martínez-Torró et al., 2020).

The three avian species occupy distinct positions along this size gradient. MG carries the largest genome (996 kb; 31% GC); goose-origin MG strains display highly variable gene counts dependent on genome annotation pipelines (Papazisi et al., 2003; Zhou et al., 2024). MG retains a broader set of central carbon metabolism genes than MS and MGn, although the completeness and *in vivo* contribution of these pathways require strain-level verification. This metabolic repertoire has been proposed to underlie its broader tissue tropism—a plausible hypothesis as a direct causal test has not been reported (Zhou et al., 2024). Structurally, the MG genome is dynamic. Indels and nonsynonymous substitutions cluster in regions encoding drug resistance determinants and surface-interaction proteins—CrmA, GapA, and VlhA. The contraction of DNA-binding protein diversity is consistent with nucleoid-associated protein-mediated regulatory rewiring (Papazisi et al., 2003; Du et al., 2025).

MS presents a different configuration. Its genome is more compact (~0.8 Mb) and structurally more stable, yet it encodes six coding sequences with no detectable ortholog in any other sequenced *Mycoplasma* species; these have been validated as diagnostic targets (Vasconcelos et al., 2005). This claim rests on a limited number of sequenced strains, and its scope should be revisited as genomic sampling expands. The broader observation it illustrates, however, is that genome reduction does not proceed uniformly. Each species retains a gene repertoire shaped by its particular ecological pressures. The boundaries of that repertoire define what the pathogen can and cannot do within the host.

MGn is included as a comparator because it is epidemiologically relevant, but current genomic and functional data are insufficient to assign it a pathogenic profile with the same confidence as MG or MS. Until 2026, MGn was represented only by a small number of incomplete draft assemblies. The first complete genome sequence (strain K285) has now been reported (Leigh and Evans, 2026). It is the most important resource for closing this gap, but it is not yet accompanied by high-quality functional annotation or a phenotypically characterized strain. Genome size, GC content, and sequence-variation hotspots therefore remain largely unexplored.

The full extent of this asymmetry is quantified in Table 1. The genomic and mechanistic differences described in this section are summarized schematically in Figure 1.

**Table 1:** Species evidence map for MG, MS, and MGn.

Species	Complete genomes (n, first reported year)	Host–pathogen transcriptomics	ML training datasets and validation strategies	Experimental validation of ML-prioritized features
MG	38, first complete 2003 (Papazisi et al., 2003, strain R(low))	Multiple *in vivo* and *in vitro* studies (Wang et al., 2022; Wang et al., 2024; Saha et al., 2025); cell line and animal infection models used; sample sizes are small and variable across studies	Pangenome matrices available (RefSeq + GenBank); AMR phenotype–genotype pairs reported; transfer learning from pre-trained models feasible; cross-validation and SHAP analyses applied	Validated: mnuA nuclease—targeted CRISPR/Cas disruption attenuated virulence in MG (Klose et al., 2022); a comparable role reported for 5′-nucleotidase in *M. bovis* (Gelgie et al., 2024) — Level A
MS	32, first complete 2005 (Vasconcelos et al., 2005, strain 53)	Synovial fibroblast transcriptomics (Liu et al., 2020); fewer multi-species comparative *in vivo* studies; sample sizes are small and variable	Pangenome matrices available; AMR phenotype–genotype pairs limited; transfer learning from MG feasible; cross-validation applied	Partial: E2 (dihydrolipoamide acetyltransferase) inferred from proteomic correlation; *in vivo* disruption pending — Level B
MGn	1, first complete 2026 (Leigh and Evans, 2026, strain K285)	None: no MGn-specific host–pathogen transcriptomic dataset exists; the studies cited for MG and MS do not extend to MGn; infection conditions are therefore not standardized	None: pangenome matrices not yet computed for ML training; no AMR phenotype–genotype pairs; ML applications to MGn do not exist in the literature	None: every mechanistic claim for MGn is computational or inferential; no ML-prioritized feature has received experimental validation — Level C

**Figure 1:**
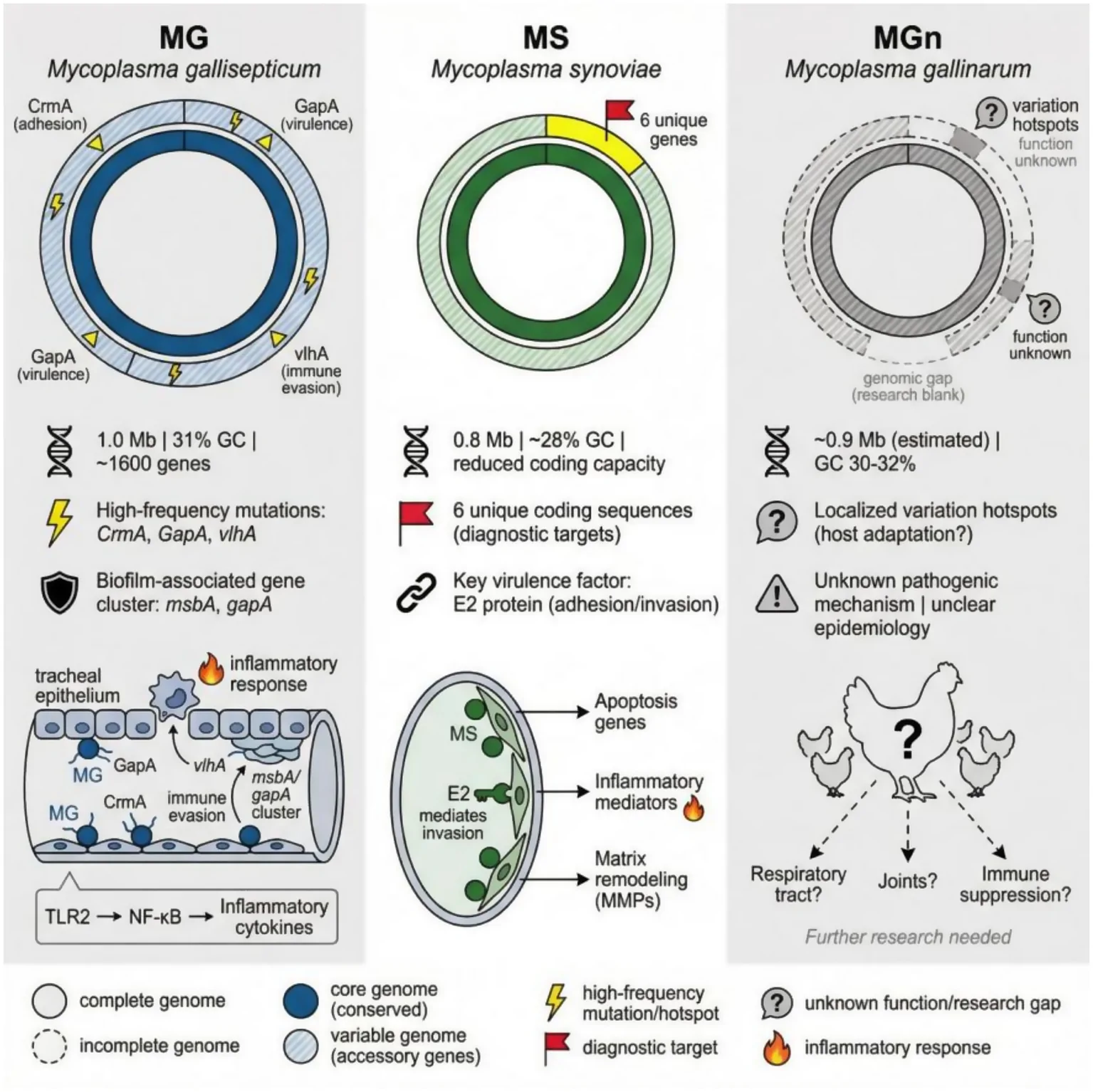
Comparative genomic architectures and representative pathogenic mechanisms of three avian *Mycoplasma* species.

Traditional multiple-sequence alignment is poorly suited to genomes shaped by recombination, phase variation, and gene gain and loss at the scale seen in *Mycoplasma* (Domingo-Sananes and McInerney, 2021). Network-based pangenomic tools address this by representing genomes as nodes in a gene-sharing graph, separating core from accessory elements and requiring no reference genome (Garrison et al., 2024). Random forest models trained on these network-derived features have identified gene co-occurrence and mutual exclusion patterns that alignment-based approaches miss (Kuronen et al., 2024). Graph convolutional networks extend this capacity to multi-omics integration, enabling subtype classification and feature prioritization directly within pangenomic feature spaces (Kuronen et al., 2024; Garrison et al., 2024).

Schematic comparison of *Mycoplasma gallisepticum* (MG), *M. synoviae* (MS), and *M. gallinarum* (MGn) based on available genomic and functional data. For each species, the upper panel represents the genome as a circular map with core genes (solid fill) and accessory genes (hatched fill); the lower panel illustrates the primary host-interaction context.

*Mycoplasma gallisepticum*: The largest genome of the three harbors mutation-enriched regions in virulence-associated loci (*CrmA*, *GapA*, and *vlhA*), reflecting elevated evolutionary pressure on surface-exposed determinants. Colonization of the tracheal epithelium is associated with TLR2/MyD88/NF-κB-mediated inflammatory responses and NLRP3 inflammasome activation.

*Mycoplasma synoviae*: The more compact genome is structurally stable but encodes six coding sequences absent from all other sequenced *Mycoplasma* species, which have been validated as species-specific diagnostic targets. Pathogenesis centers on synovial fibroblast interactions, with documented reprogramming of apoptotic, inflammatory, and matrix remodeling programs.

*Mycoplasma gallinarum*: Genomic characterization is incomplete; available draft assemblies indicate an intermediate genome size, but functional annotation of candidate virulence loci and host-interaction data remain insufficient for mechanistic inference. This species represents the primary knowledge gap in the comparative framework.

Abbreviations: core, genes conserved across all strains of a species; accessory, genes present in a subset of strains; TLR2, toll-like receptor 2; NF-κB, nuclear factor kappa B; NLRP3, NLR family pyrin domain-containing protein 3.

Genome counts were obtained from NCBI Datasets (RefSeq), using the assembly level “Complete genome,” accessed in August 2026. Taxonomy follows the NCBI 2024–2025 reclassification of Mollicutes: *Mycoplasmoides gallisepticum, Mycoplasmopsis synoviae,* and *Mycoplasmopsis gallinarum* (revised from *Mycoplasma*). Empty and single-entry cells are shown as recorded, following PRISMA 2020 transparency principles (Page et al., 2021). Sample sizes for transcriptomic studies and ML training-set sizes are stated qualitatively because no dedicated ML study on avian *mycoplasmas* exists.

### 2.2 Virulence factor annotation and validation

Pangenome analysis can help narrow the number of genes that require functional investigation. In avian *Mycoplasma*, comparative genomic studies have identified gene families enriched in pathogenic lineages, conserved among virulent strains but absent from attenuated derivatives, or associated with signatures of positive selection (Tulman et al., 2012). As the amount and type of available evidence differ substantially among MG, MS, and MGn, we consider their pathogenic features in four areas: Adhesion and colonization, tissue tropism, immune modulation, and antigenic variation. We indicate whether each observation is supported by functional experiments, omics data, comparative analysis, or more limited evidence.

#### 2.2.1 Adhesion and colonization

Experimental evidence for attachment to the respiratory epithelium is strongest for MG. GapA and CrmA, together with the phase-variable VlhA lipoprotein family, contribute to the colonization of the respiratory tract (Tulman et al., 2012; Pflaum et al., 2020). Clear orthologs of these MG adhesins have not been identified in MS or MGn. Their absence, however, does not demonstrate reduced adhesive capacity because different *Mycoplasma* species may rely on distinct adhesin families (Barbosa et al., 2022; Liu et al., 2024). In MGn, proteomic studies have identified putative cytadhesin homologs and an expanded aminopeptidase repertoire that may contribute to nutrient acquisition (Wan et al., 2010). In a survey of 299 isolates, MGn formed weaker biofilms *in vitro* than MG and MS (Catania et al., 2024). This difference may reflect variation in colonization-related traits, but it should not be interpreted as direct evidence that MGn is non-pathogenic.

#### 2.2.2 Tissue tropism

MG is principally associated with infection of the respiratory tract, although the molecular determinants of its tissue distribution remain incompletely understood (Papazisi et al., 2003; Zhou et al., 2024). MS is associated with respiratory infection, as well as synovial and systemic disease, with synovial involvement being a clinically important feature of infection (Liu et al., 2020). MGn has a broad host range, and proteome-level inference has tied this breadth to genes implicated in host adaptation (Wan et al., 2010); the basis of its tissue tropism within avian hosts, however, remains unclear.

#### 2.2.3 Immune modulation

MG induces a pro-inflammatory response involving TLR2/MyD88/NF-κB signaling and NLRP3-related macrophage responses. Tissue injury may therefore result from both bacterial activity and host inflammatory activation (Wang et al., 2023; Wang et al., 2024). In MS, infection of synovial fibroblasts is associated with changes in apoptotic, inflammatory, and matrix remodeling pathways. Dihydrolipoamide acetyltransferase (E2) has been identified as a virulence-associated factor in experimental studies, and other studies suggest that MS may interfere with antiviral interferon signaling (Liu et al., 2020). For MGn, a relatively limited humoral response has been reported, although the available evidence is sparse (Wan et al., 2010). No dedicated MGn transcriptomic dataset is yet available; putative oxidative- and ER stress-related signatures have been inferred mainly from small-scale or indirect studies and require confirmation in controlled infection experiments.

#### 2.2.4 Antigenic variation

In MG, the VlhA, pMGA, and PvpA surface lipoproteins undergo phase variation, which may link antigenic diversification with immune evasion and epithelial attachment (Markham et al., 1994; Tulman et al., 2012; Pflaum et al., 2020; Fatideh et al., 2022). Antigenic variation involving the vlhA family has also been reported in MS, although its functional characterization remains less extensive than that of MG. No antigenic variation system has yet been characterized in MGn, and this aspect of MGn biology remains unresolved.

#### 2.2.5 Strength of the supporting evidence

The three species are not supported by equivalent levels of evidence. For MG, mechanistic conclusions are supported by targeted genetic manipulation, including mnuA disruption using an endogenous CRISPR/Cas system, together with studies of host-signaling pathways (Chen et al., 2021; Klose et al., 2022; Evsyutina et al., 2022). Evidence for MS relies more heavily on cell culture, transcriptomic, and proteomic studies, with fewer comparable targeted *in vivo* validations (Liu et al., 2020). For MGn, most mechanistic interpretations remain based on comparative genomics, proteomics, or limited transcriptomic data. The recent expansion of MGn genomic resources may support further investigation, but its functional evidence base is still less developed than those available for MG and MS (Leigh and Evans, 2026). Table 1 quantifies this asymmetry at a glance.

The following sections examine these features in greater detail. Throughout the review, experimentally established mechanisms are distinguished from findings based mainly on omics data or comparative inference.

### 2.3 Horizontal gene transfer and antimicrobial resistance evolution

Genomic diversity in avian *mycoplasmas* is not driven solely by mutation and vertical inheritance. Horizontal gene transfer (HGT) has contributed to these genomes, enabling exchange among co-colonizing strains and introducing metabolic and virulence-associated functions into the accessory gene pool (Citti et al., 2018; Dordet-Frisoni et al., 2014, 2019). In organisms with minimal genomes and limited functional redundancy, lateral acquisition is in principle a faster route to novel function than *de novo* evolution. However, direct experimental evidence for this inference in avian *mycoplasmas* specifically remains limited (Citti et al., 2018).

Antimicrobial resistance (AMR) gene architecture reflects HGT history. In MG, resistance determinants are concentrated in the core genome, consistent with stable, vertically inherited resistance that is relatively amenable to sequence-based prediction (Kachabi et al., 2025). In MS, resistance is more heterogeneous: Mutations associated with decreased susceptibility to fluoroquinolones, macrolides, and lincomycin have been characterized (Bekő et al., 2020), and a greater contribution of the accessory genome to resistance variation has been reported (Forero-Marin et al., 2026). The architecture of AMR in MGn remains insufficiently sampled (Kachabi et al., 2025). Phylogenetic analyses using mgc2 and 16S rRNA markers have traced transmission networks across commercial poultry systems and documented repeated positive selection at resistance-associated loci, particularly drug target genes and efflux pump sequences (Bekő et al., 2020; Forero-Marin et al., 2026). The broader implication is that AMR and virulence evolution may be functionally coupled. Mobile elements can link resistance and host-interaction traits in some *mycoplasmas*. The extent of this coupling in avian species, however, remains unresolved (Citti et al., 2020; Bekő et al., 2020). Antimicrobial resistance is therefore addressed in this dedicated section to keep evidence of resistance and virulence distinct, and their potential coupling is revisited in the comparative synthesis (Section 3). This structural separation should not be read as an assumption that the two are independent.

## 3 Machine learning applied to divergent pathogenic mechanisms

### 3.1 What machine learning contributes to *Mycoplasma* research

Machine learning applications in avian *mycoplasma* research are constrained by the data available. The field lacks large, harmonized datasets. *Mycoplasma* genomes are compact, regulatory networks are reduced, and experimentally matched comparisons across species, strains, and infection models remain scarce. These constraints do not make ML uninformative, but they define the class of questions it can currently address reliably. In this context, ML is best understood as a method for prioritizing testable hypotheses from fragmented genomic, transcriptomic, and phenotypic data, not as a substitute for the mechanistic experiments it is meant to guide.

The most tractable application is identification and classification. Given a genomic sequence, gene-presence matrix, or molecular feature set, classifiers can assign species identity, strain group, or antimicrobial resistance category. Random forest and support vector machine models built on pangenomic presence–absence matrices have been used to predict AMR phenotypes and identify resistance-associated genomic features across bacterial species (Do et al., 2024; Kim et al., 2022). These tasks are relatively well-bounded: The feature space is defined, and the outcomes are categorical. Performance can improve when genomic data are combined with epidemiological variables such as flock type, vaccination history, or antimicrobial exposure history. This finding suggests that resistance phenotypes are not fully captured by genome sequence alone and that epidemiological context contributes to prediction (Do et al., 2024).

Prioritization addresses a harder question: Not whether a model can classify strains, but which genes or variants merit experimental follow-up. Random forest importance scores, Least Absolute Shrinkage and Selection Operator (LASSO) regression, and Boruta feature selection applied to pangenomic datasets can identify candidate virulence-associated genes whose presence, absence, or sequence variation correlates with disease severity or host range phenotype (Do et al., 2024). This approach carries a specific hazard: Virulence is not a single-gene trait, and field isolates typically differ at many linked loci simultaneously. A highly ranked feature may be causal, may tag a broader genomic background, or may reflect shared geography or population structure rather than biology. Feature selection should therefore be treated as candidate prioritization, not as mechanistic evidence.

Network-based inference addresses a third problem. Weighted gene co-expression network analysis (WGCNA) organizes transcriptomic data into co-expression modules and associates those modules with quantitative traits—lesion scores, bacterial load, immune markers, or tissue-specific responses (Langfelder and Horvath, 2008). For MG and MS, disease reflects both bacterial factors and host tissue responses. Co-expression analysis can identify host modules activated during infection and compare them across species. This can suggest inflammatory, apoptotic, or matrix remodeling programs that may be species-specific (Wang et al., 2024; Liu et al., 2020). The limitation here is fundamental: Correlation structure is not causal regulation. A module associated with severe disease may contain drivers, downstream responders, and unrelated genes that co-vary because of cell composition shifts rather than shared regulation. Causal claims require independent perturbation.

Identification, prioritization, and network inference require different algorithm classes and different evidence standards. A recurring weakness in the literature—not specific to *mycoplasma* research—is to begin with an algorithm and retrospectively identify a biological question that fits it. A more productive approach is to define the biological problem first and then select the model class and validation strategy that the available data can support.

### 3.2 From computational prediction to biological discovery

The value of ML in *mycoplasma* research ultimately depends on whether computational predictions lead to experimentally supported biological findings. By that standard, convincing examples remain limited, and acknowledging this matters: It defines both the current contribution of ML and the limits of the approach.

The clearest example involves the mnuA nuclease gene in MG. Feature prioritization methods applied to a pangenomic matrix of approximately 1,500 orthogroups ranked mnuA among candidate genes associated with a pathogenic phenotype. Targeted disruption of the gene using the endogenous CRISPR/Cas system subsequently impaired bacterial survival and attenuated pathogenicity in infection experiments (Klose et al., 2022). A comparable role has been reported for a 5’-nucleotidase in *Mycoplasma bovis* (Gelgie et al., 2024). The sequence—genome-wide screening, explicit hypothesis generation, genetic perturbation, and phenotype testing—provides a coherent link between computational prioritization and biological inference. The important caveat is that this closed prediction-to-validation loop is the exception, rather than the routine workflow, in avian *mycoplasma* research.

A second line of evidence concerns tissue-dependent vlhA expression in MG. Transcriptomic analyses of infected tracheal and conjunctival tissues are consistent with active surface remodeling during infection, with distinct vlhA variants dominating in different tissue environments (Pflaum et al., 2020; Liu et al., 2024). This observation refines a mechanistically important hypothesis. However, individual vlhA variants have not been systematically perturbed and tested for effects on tropism, immune evasion, or disease outcomes. The result remains largely associational and should not be treated with the same evidentiary weight as the mnuA disruption study.

These two examples illustrate a practical division of labor. Computational methods can screen large numbers of genes, strains, or host response features more efficiently than manual evaluation and can detect combinations that single-feature comparisons would miss. Experimental validation remains the decisive step. CRISPR/Cas and CRISPRi platforms in *mycoplasmas* are valuable precisely because they allow ranked candidates to be tested in the organisms that generated the predictions (Klose et al., 2022; Evsyutina et al., 2022). The limiting factor is no longer the capacity to generate candidates but the throughput of functional testing in technically demanding organisms.

Failed predictions are rarely reported. Some ML-prioritized candidates have not been validated—either because the predicted gene was not expressed under infection conditions, because disruption produced no measurable phenotype, or because the apparent association reflected a linked genomic region rather than the candidate itself (Do et al., 2024). Without these outcomes in the public record, the false-positive rate of ML-based prioritization in avian *mycoplasmas* cannot be estimated, and calibration of future models is impaired. Transparent reporting of candidate selection criteria, validation attempts, and negative results would be more informative than additional models reporting only internal cross-validation metrics. This prediction-to-validation sequence is illustrated in Figure 2.

**Figure 2:**
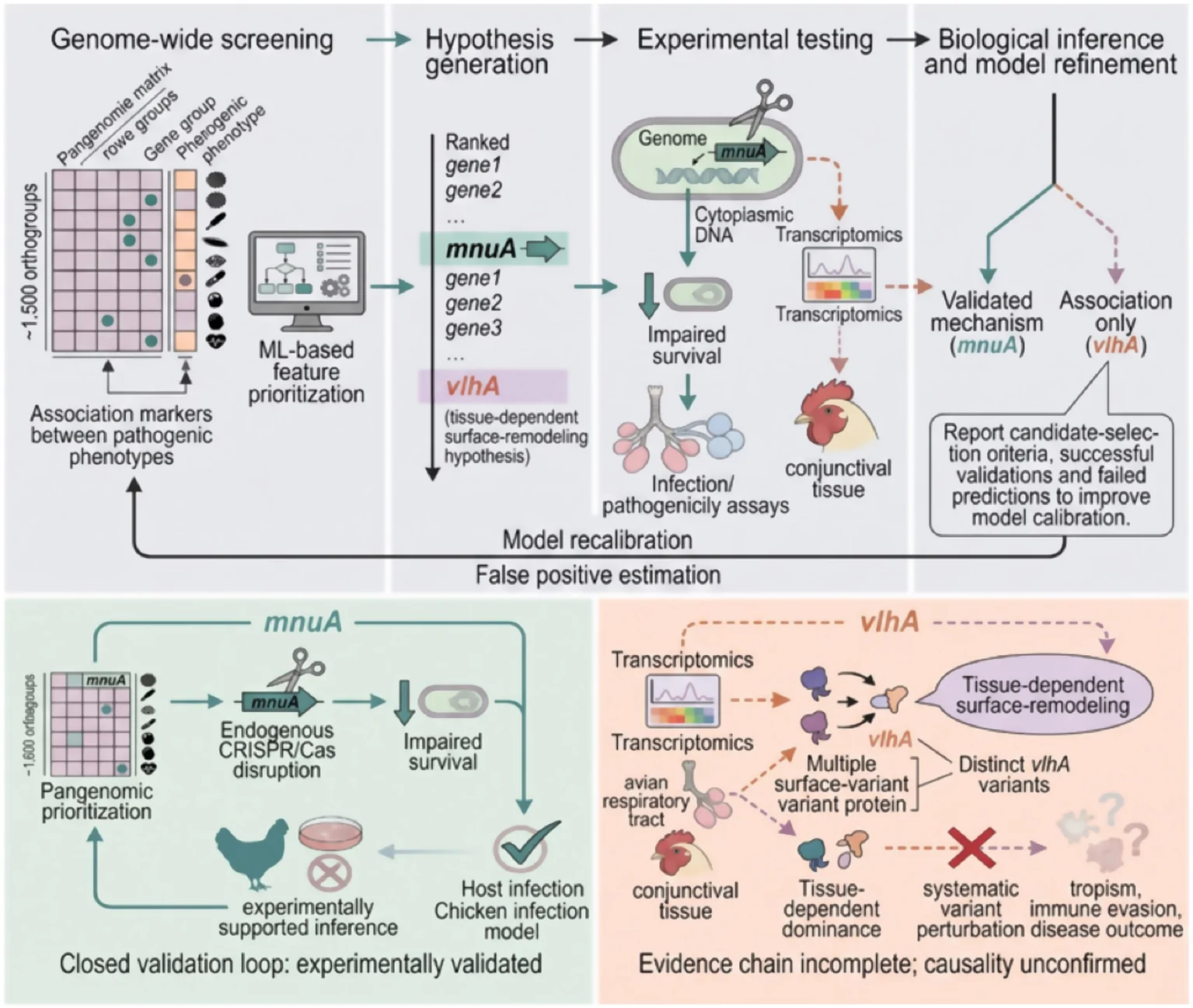
From computational prediction to biological discovery in avian *Mycoplasma*.

ML-based analysis of pangenomic data prioritizes candidate genes and generates testable pathogenicity hypotheses. The mnuA pathway represents a closed validation loop, in which candidate prioritization was followed by CRISPR/Cas-mediated disruption, impaired bacterial survival, and attenuated pathogenicity in chicken infection experiments. In contrast, tissue-specific transcriptomic analysis identifies distinct vlhA variants and supports a tissue-dependent surface remodeling hypothesis, but causal effects on tropism, immune evasion, and disease outcomes remain unconfirmed because systematic variant perturbation has not been performed. Reporting both successful and failed validations is essential for estimating false-positive rates and improving model calibration. The mnuA closed-loop schematic is conceptualized based on the endogenous CRISPR/Cas-mediated mnuA disruption and chicken infection experiments reported by Klose et al. (2022); all other elements are illustrative.

### 3.3 Structural bottlenecks: validation and interpretability

In total, two structural bottlenecks limit the application of machine learning in this field: Validation and interpretability. On the validation side, accuracy alone is an insufficient reporting metric; sensitivity, specificity, and calibration across classes should accompany it because overall accuracy can conceal poor performance on the resistant or virulent strains that matter most clinically.

Interpretability raises a separate problem. The most accurate architectures, such as gradient-boosted trees, neural networks, and ensemble methods, are often opaque. This opacity may be acceptable when a model is used only for screening, but when it informs veterinary diagnostic or treatment decisions, the biological basis of a prediction matters. SHapley Additive exPlanations (SHAP) analyses of XGBoost models for *mycoplasma* resistance and virulence suggest that a small number of genes or SNPs carry most of the predictive signal (Kim et al., 2022; Do et al., 2024). If this result holds in independent datasets, simpler models, such as sparse logistic regression or shallow decision trees, may achieve comparable accuracy while pinpointing the specific genes or SNPs responsible and should be compared systematically against more complex alternatives.

### 3.4 Practical strategies for the next phase

The practical question is how to build useful models from a few dozen genomes rather than the tens of thousands available in other pathogen fields. Table 1 makes the scale concrete. Complete genomes number in the dozens for MG, fewer for MS, and, until 2026, none were available for MGn. Avian *mycoplasma* research therefore operates in a few-shot regime, where data-hungry architectures are poorly matched to the problem. Genome architecture compounds the constraint. At under roughly 1 Mb, these genomes carry small gene repertoires, so pangenome presence/absence matrices are low-dimensional but sparse and phylogenetically confounded, as neighboring strains share most of their content and thus inflate apparent associations. This structure favors regularized and tree-aware models over deep networks trained from scratch.

Domain shift adds a third obstacle, and it operates at three levels. The first is between host tissues: A model trained on tracheal samples will not necessarily transfer to synovial tissue. The second is between strains with different passage histories. The third, and most consequential, is between species, where a model built on MG features cannot be assumed to transfer to MGn. This is the same asymmetry that runs throughout the comparative framework presented in Section 3.

#### 3.4.1 Foundation models and transfer learning

Genomic foundation models offer a partial answer. The Nucleotide Transformer family, pre-trained on 850 diverse genomes, predicts accurately in low-data settings and can be fine-tuned at modest cost (Dalla-Torre et al., 2025). DNABERT-2 provides a multi-species model with a standardized benchmark that includes bacterial species classification (Zhou et al., 2024). Evo, trained on 2.7 million prokaryotic and phage genomes at single-nucleotide resolution, supports zero-shot function prediction across DNA, RNA, and protein sequences (Nguyen et al., 2024). The caution is that these models are uneven: Benchmark performance varies substantially by task, and specialized models still outperform foundation models on several genomic tasks (Feng et al., 2025). For avian *mycoplasmas,* the realistic path is therefore to transfer learning from a pre-trained model followed by limited fine-tuning, rather than training from scratch.

In addition, two further design choices follow from the data. Class imbalance requires more than routine oversampling; cost-sensitive learning, which penalizes minority class errors, adjusts the decision boundary without fabricating observations, and anomaly detection frameworks may suit rare events such as newly emerging resistance patterns. Benchmarking simple and complex models on the same data would clarify how much signal is actually present. If a compact feature set carries most of it, sparse logistic regression or a shallow tree may match XGBoost or a deep network at lower cost and with clearer interpretability. No published study has applied machine learning directly to avian *mycoplasmas*, so this comparison remains untested. The closest relevant work comes from adjacent bacterial systems: PanKA, which predicts antimicrobial resistance from population pangenomes (Do et al., 2024); VirulentHunter, a deep learning model for virulence factor prediction (Chen et al., 2025); and a clinical critique of ML-based AMR prediction that highlights the gap between benchmark accuracy and clinical utility (Kim et al., 2022).

This absence is itself a finding, and it is the concrete opportunity toward which Sections 3 and 4 build. Validation evidence categories also need clearer definition—a computational association should be labeled a candidate, a replicated association a stronger candidate, and a disrupted and phenotyped gene mechanistically supported. All of this depends on shared data. Open, consistently annotated genomic and phenotypic datasets, with agreed metadata fields, would improve reproducibility and enable the cross-study comparisons discussed above. Federated learning may eventually allow joint training without transferring raw sequence data (Rieke et al., 2020). Across these considerations, a consistent workflow emerges: pangenome construction, feature encoding, candidate ranking, validation on independent data, targeted functional testing in cell and infection models, and model iteration (Figure 2).

## 4 Pangenomic and transcriptomic evidence for divergent pathogenic mechanisms

### 4.1 Metabolic pathway divergence and host resource exploitation

The metabolic constraint on *mycoplasma* pathogenicity is direct: Near-complete loss of biosynthetic capacity means that a competitive advantage in extracting or processing host metabolites can translate into a colonization advantage (Citti et al., 2018; Martínez-Torró et al., 2020). Pangenomic reconstructions indicate that MG, MS, and MGn resolve this shared constraint through different metabolic configurations.

MG retains the most complete central carbon metabolism of the three species. Key glycolytic enzymes (pyruvate kinase, lactate dehydrogenase, and phosphofructokinase) are present and transcriptionally active during infection (Mahdizadeh et al., 2021; Lu et al., 2023). This metabolic completeness has been proposed to underlie MG’s capacity to exploit a wider range of host carbon sources and sustain colonization across diverse tissue environments. This is consistent with its broader tissue tropism and more pronounced inflammatory pathology (Wang et al., 2022). In MS and MGn, several orthologs of these enzymes show evidence of gene decay or consistent transcriptional downregulation, suggesting greater dependence on host-derived nutrients and correspondingly narrower substrate flexibility (Vasconcelos et al., 2005; Liu et al., 2024). Whether metabolic differences are a cause or a downstream consequence of niche specialization has not been resolved experimentally; the correlation is consistent across independently collected strain panels, but the available evidence supports no more than a correlation.

Biofilm formation follows a parallel gradient. MG produces robust biofilms under standard culture conditions; MS and MGn form weaker or structurally inconsistent biofilms under the same conditions (Catania et al., 2024). The regulatory connections between metabolic flux and biofilm matrix production have not yet been characterized in any avian *mycoplasma*, but the pattern is consistent with the idea that a more complete metabolic network can sustain the energetic demands of biofilm architecture. Whether this gradient has *in vivo* relevance—and whether biofilm capacity contributes to colonization persistence or antimicrobial tolerance in the avian host—remains untested.

Metabolic state and immune responses intersect in ways that have been partially characterized for MG. AMPK, a central regulator of cellular energy homeostasis, modulates inflammation through downstream SIRT1-mediated signaling during MG infection; quercetin’s observed suppression of MG-induced inflammatory injury appears to operate through this AMPK/SIRT1/NF-κB axis (Lu et al., 2023). This identifies the pathway as engaged during infection. It does not establish the endogenous contribution of AMPK/SIRT1/NF-κB signaling to disease progression independently of pharmacological intervention—a distinction that matters for interpreting therapeutic potential. Whether MS or MGn engage this metabolic-immune interface at all, or activate different nodes in the same network, has not been examined in any direct comparative experiment.

### 4.2 Adhesion and invasion: genomic predictions and evidential limitations

Adhesion to the host epithelium is an initiating step in *mycoplasma* infection, and the molecular basis is better characterized in MG than in any other avian species. GapA, CrmA, and the VlhA lipoprotein family collectively mediate attachment to the respiratory epithelium. Individual VlhA variants show tissue-specific expression in tracheal versus conjunctival environments. MG therefore actively remodels its surface-exposed adhesin repertoire during infection rather than expressing a fixed set of attachment proteins (Tulman et al., 2012; Pflaum et al., 2020). This dynamic is mechanistically important: The same molecular machinery that enables colonization across multiple tissue sites simultaneously drives immune evasion through antigen variation, making adhesion and immune escape functionally inseparable in MG.

The comparative picture for MS and MGn is substantially less complete, and the available evidence must be described precisely—what it shows and what it does not.

Genomic surveys indicate that MS and MGn lack clear orthologs of MG adhesins, including GapA and several VlhA family members (Barbosa et al., 2022; Liu et al., 2024). This finding is consistent with an altered host cell tropism—MS colonizes synovial tissue rather than the respiratory epithelium, and the absence of MG-type respiratory adhesins is congruent with that difference. However, the absence of a genomic ortholog establishes only that the same protein is not present. *Mycoplasmas* are capable of convergent adhesin evolution, and related species, including *M. pirum*, *M. genitalium*, and *M. agalactiae*, have evolved distinct, species-specific adhesin families that perform analogous attachment functions through different protein architectures (Barbosa et al., 2022; Tham et al., 1994; Reddy et al., 1995). It is therefore incorrect to infer from ortholog absence alone that MS or MGn have reduced adhesion capacity; what can be inferred is that they use different molecular solutions for attachment, whose identities remain largely uncharacterized.

Comparative genomic evidence further suggests that MS and MGn may have lost or pseudogenized certain invasion-associated genes present in MG. This could account for observed differences in intracellular invasion and tissue dissemination capacity (Vasconcelos et al., 2005; Liu et al., 2024). This is a reasonable hypothesis, but it has not been tested by gene disruption or complementation in any of the three species and should be treated as a candidate explanation rather than an established mechanism.

This gap is wider than in any other dimension of the comparative framework. The experiment that would close it most directly is a single systematic study applying the same platform (pangenomic candidate prediction, *in vitro* adhesion and invasion assays in relevant avian cell lines, and CRISPRi-based disruption of predicted adhesins) to MG, MS, and MGn in parallel. Until such work is completed, comparative adhesion claims rest on genomic inference, and the mechanistic account of how these three species colonize distinct tissue compartments remains incomplete.

### 4.3 Immune evasion and its Interface with antimicrobial resistance

The immune evasion strategies of the three species differ qualitatively. The transcriptomic evidence for these differences is more informative than that available for adhesion. This is partly because host immune responses leave a measurable footprint in gene expression data, which can be collected without the direct manipulation experiments that adhesion studies require.

MG infection activates TLR2/MyD88/NF-κB signaling and generates a strong pro-inflammatory response characterized by elevated IL-1β, TNF-α, and IL-6 (Wang et al., 2022; Wang et al., 2024). NLRP3 inflammasome activation drives M1 macrophage polarization, amplifying tissue injury (Wang et al., 2024). The transcriptomic picture is of a pathogen whose pathology arises substantially from the inflammatory response it provokes, so tissue damage is a direct consequence of host immune activation rather than a side effect of bacterial growth. This distinction has therapeutic implications: Suppressing the host response without eliminating the infection may impose additional risks (Wang et al., 2024; Liu et al., 2024).

MS operates through a mechanistically different mode. Infection of synovial fibroblasts reprograms transcriptional programs governing apoptosis, matrix remodeling, and inflammatory signaling, with the E2 virulence factor identified as a central contributor to this reprogramming (Liu et al., 2020; Yin et al., 2025). Available transcriptomic data also indicate disruption of antiviral interferon signaling pathways in MS-infected cells. This positions MS as a pathogen that interferes with specific host signal transduction programs in a specialized tissue compartment, mechanistically distinct from the broad inflammatory activation that characterizes MG infection.

MGn data are sparse and carry a specific interpretive caveat. Currently available transcriptomic datasets are small, and infection conditions are not standardized across studies. It is therefore not possible to determine whether the reported mixed inflammatory, autophagic, and antioxidant signatures reflect genuine mechanistic diversity or the higher noise inherent in poorly characterized, underpowered experiments. Controlled infection experiments using well-characterized MGn strains in a defined avian host model, ideally under conditions comparable to those used for MG and MS, would generate comparative data of substantially greater interpretive value than additional single-species transcriptomic surveys. Table 2 annotates each mechanistic claim with its evidence level. The comparative intracellular signaling cascades discussed in this section are summarized in Figure 3.

**Table 2:** Comparative pathogenic profiles of MG, MS, and MGn.

Dimension	MG	MS	MGn
Metabolic strategy	Complete central metabolism; broad substrate use	Streamlined; niche-specialized	Incomplete data; appears intermediate
Adhesion/invasion	Multi-locus adhesin repertoire; tissue-remodeling VlhA proteins	Distinct adhesin set; synovial tropism; molecular identity largely uncharacterized	Largely uncharacterized
Immune evasion mode	Inflammation-driven (TLR2/NF-κB, NLRP3)	Antiviral pathway disruption; interferon signaling interference	Mixed signals; inflammation, autophagy, antioxidant response
Host stress response	Oxidative stress; Nrf2-dependent defense	ER stress; matrix remodeling	Poorly resolved
AMR architecture	Core genome; stable vertical inheritance	Accessory genome; HGT-driven	Accessory genome; HGT-driven (limited data)
Evidence confidence	A: multiple functional studies	B: fewer but internally consistent	C: genomic inference, limited experiments

**Figure 3:**
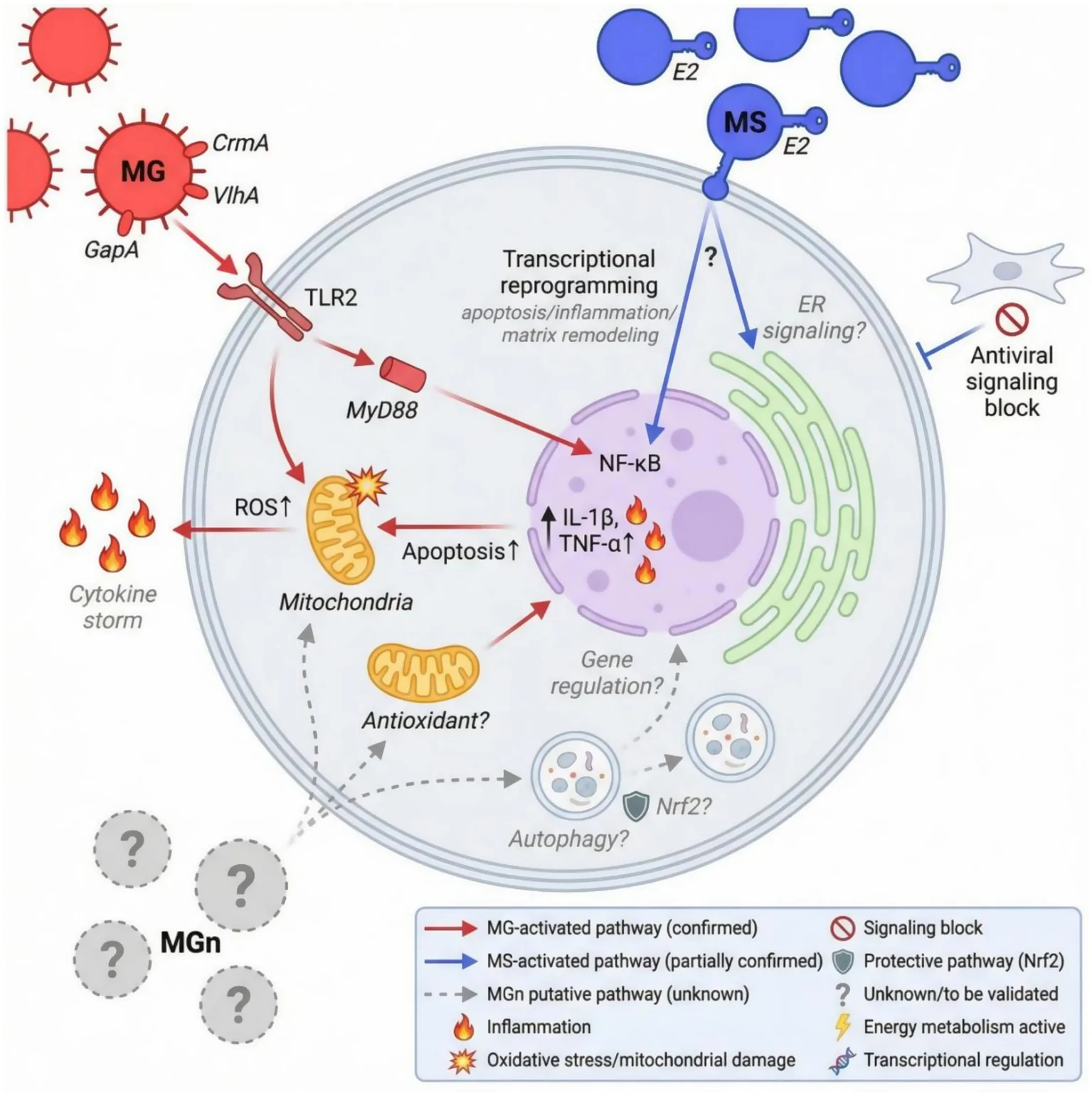
Comparative intracellular pathogenic signaling of *Mycoplasma gallisepticum* (MG), *M. synoviae* (MS), and *M. gallinarum* (MGn) in avian host cells.

Immune evasion and antimicrobial resistance are distinct but potentially interconnected processes in these species. Biofilms can impede immune clearance and reduce antimicrobial susceptibility through matrix-associated diffusion barriers, altered metabolic activity, and physiological heterogeneity among embedded cells (Flemming and Wingender, 2010). In avian *Mycoplasma*, however, evidence for biofilm formation is currently limited to *in vitro* studies. MG forms robust biofilms under standard culture conditions, whereas MS and MGn exhibit weaker or less consistent biofilm formation (Catania et al., 2024; Section 3.1). Whether these differences affect antimicrobial susceptibility or persistence in the avian host remains unknown. Immune evasion may also promote persistent infection and increase cumulative antimicrobial exposure, thereby creating conditions that favor the selection or maintenance of resistant populations (La Rosa et al., 2022). The genomic determinants of antimicrobial resistance are discussed separately in Section 1.3.

Arrows and pathway elements are color-coded by evidence confidence: Red solid arrows indicate experimentally confirmed MG-activated pathways, blue solid arrows indicate MS-activated pathways with partial experimental support, and gray dashed arrows indicate putative MGn pathways inferred from genomic or indirect evidence without direct experimental validation. Question marks denote molecular connections or outcomes that remain to be validated.

MG (red) initiates infection via surface adhesins, including GapA, CrmA, and VlhA, engaging host TLR2 and the downstream adaptor MyD88. This activates NF-κB-mediated transcriptional reprogramming, driving upregulation of pro-inflammatory cytokines IL-1β and TNF-α, mitochondrial dysfunction, reactive oxygen species (ROS) accumulation, and apoptosis induction. The combined effect contributes to cytokine storm in susceptible birds. MG pathways shown represent the most thoroughly characterized signaling cascade among the three species reviewed.

MS (blue) induces synovial fibroblast reprogramming through dihydrolipoamide acetyltransferase (E2), which has been identified as a virulence-associated factor. The downstream response involves NF-κB activation and endoplasmic reticulum stress, whereas suppression of host antiviral interferon signaling may facilitate immune evasion. The MS pathway is only partially characterized; the relevant receptor interactions and the complete downstream cascade remain to be defined.

MGn (gray dashed) pathogenic mechanisms are largely uncharacterized. Putative involvement of gene regulation, autophagy induction, antioxidant responses, and Nrf2-mediated cytoprotection is indicated based on genomic inference and limited indirect evidence, but no confirmed intracellular signaling cascade was established for MGn at the time of this review. The Nrf2 protective pathway (shield symbol) represents a host-encoded cytoprotective response implicated in the context of MG-induced oxidative stress; whether this pathway is similarly engaged during MS or MGn infection has not been determined.

Abbreviations: TLR2, toll-like receptor 2; MyD88, myeloid differentiation primary response protein 88; NF-κB, nuclear factor kappa B; IL-1β, interleukin-1 beta; TNF-α, tumor necrosis factor alpha; ROS, reactive oxygen species; ER, endoplasmic reticulum; Nrf2, nuclear factor erythroid 2-related factor 2; E2, dihydrolipoamide acetyltransferase E2; GapA, cytadherence-associated protein a; CrmA, cytadherence-related membrane protein a; VlhA, variable lipoprotein and hemagglutinin a.

### 4.4 Differential host stress response profile

Each of the three *avian mycoplasma* species elicits a distinct host stress response profile, and these profiles map onto the pathogenic strategies described in the preceding section.

MG infection depletes antioxidant capacity in the avian spleen and thymus and induces systemic oxidative stress, mitochondrial dysfunction, and upregulation of pro-apoptotic gene programs (Wang et al., 2023; Wu et al., 2021; Hu et al., 2021). The TLR2/NF-κB inflammatory response and oxidative stress are mechanistically coupled in this context: Inflammation generates reactive oxygen species, and the resulting oxidative damage further amplifies tissue injury in a self-reinforcing cycle (Wang et al., 2023). The Nrf2 pathway, which coordinates antioxidant enzyme expression, operates as a critical host defense node against this damage; its activation mitigates MG-induced oxidative injury, and evidence from infection models associates its suppression with more severe disease (Saha et al., 2025). Therefore, Nrf2 sits at the intersection of host protection and pathological tissue damage during MG infection. This position makes it a candidate intervention target. The caveat is that its role has been characterized mainly through pharmacological activation rather than genetic perturbation in the avian host. Hence, Nrf2 is a preclinical candidate whose clinical translation awaits *in vivo* genetic validation.

For comparative context, *M. bovis* infection in bovine macrophages engages a qualitatively different stress program, dominated by ER stress through the PERK/CHOP pathway and IL-17 signaling, rather than the TLR2-driven oxidative stress profile of MG (Zhang et al., 2022; Wu et al., 2021). The point of this contrast is that stress pathway engagement is not conserved even within the genus *Mycoplasma*. Knowledge of how one species manipulates host stress responses therefore provides a limited basis for predicting how another will behave, even when the host is taxonomically similar and the pathogen genome is structurally comparable. For MS, available transcriptomic evidence indicates a predominantly ER stress and matrix remodeling signature in infected synovial fibroblasts, consistent with the joint pathology that defines MS disease in the field (Liu et al., 2020). For MGn, transcriptomic profiles suggest concurrent engagement of oxidative and ER stress pathways, but sample sizes remain small and these results should be treated as preliminary pending replication in controlled infection experiments.

Stress response data therefore show that the infecting species shapes both the magnitude and the qualitative features of the host response. This distinction has direct implications for intervention design. A strategy targeting Nrf2-mediated oxidative pathology (appropriate for MG-associated disease) would have no predicted benefit, and could be counterproductive, in MS or MGn infections driven by distinct stress axes. Species-specific target identification is therefore necessary, not merely a refinement of therapeutic strategy.

### 4.5 Comparative synthesis: three species-specific pathogenic profiles

The evidence across metabolism, adhesion and invasion, immune modulation, host stress responses, and antimicrobial resistance architecture is summarized in Table 2. These findings indicate that MG, MS, and MGn should not be treated as minor variants of a common avian *mycoplasma* strategy. They share the constraints of genome reduction and obligate host dependence, but the available data consistently point to different patterns of host adaptation and disease expression.

MG is the best-characterized of the three. Functional studies link its adhesion machinery, TLR2/MyD88/NF-κB inflammatory signaling, NLRP3-associated macrophage polarization, oxidative tissue damage, and specific virulence-associated genes to measurable disease phenotypes. The mechanistic account is incomplete but grounded in experimental data across multiple independent research groups. MS is supported by a smaller but relatively coherent body of evidence—particularly for synovial fibroblast reprogramming, matrix remodeling, ER stress, and interferon signaling interference—that is internally consistent even if it lacks the breadth of the MG literature. MGn is the weakest point of the comparison. Most claims about its pathogenic strategy are still inferred from incomplete genomic resources and limited, underpowered transcriptomic datasets. The mixed host response pattern currently attributed to MGn may reflect genuine biological diversity, or it may reflect the noise of poorly controlled experiments with a pathogen that has not yet been adequately characterized in any experimental model.

Within these evidence limits, MG can be described as a broadly colonizing, inflammation-associated respiratory pathogen: Metabolically versatile, phase-variable at the surface, and driving pathology through sustained TLR2/NF-κB activation and oxidative tissue injury. MS is more niche-specialized, with evidence pointing to synovial adaptation, altered fibroblast transcriptional programs, and targeted interference with antiviral signaling rather than broad inflammatory provocation. MGn cannot yet be assigned a well-supported pathogenic profile. What the current data permit is a structured account of where the evidence is sufficient, suggestive, or absent, and that account is itself informative. Table 1 quantifies this asymmetry across genomes, transcriptomes, and ML training data.

Evidence levels: A, direct experimental evidence (targeted loss- or gain-of-function studies, defined infection models, or pathway inhibition); B, correlative and cell model evidence (transcriptomic/proteomic profiling or *in vitro* models without targeted *in vivo* confirmation); C, computational or inferential evidence (comparative genomics, pangenome inference, or ML prioritization). Adapted from GRADE (Guyatt et al., 2008).

## 5 Conclusion and future perspectives

This review argues that MG, MS, and MGn represent distinct pathogenic strategies, not variants of a common phenotype, although the evidence for this conclusion is graded rather than uniform (Levels A–C; Table 2). MG is a metabolically versatile, inflammation-associated respiratory pathogen whose pathology is driven largely by TLR2/NF-κB- and NLRP3-mediated host responses—a conclusion resting on direct experimental evidence (Level A). MS is more niche-specialized, with synovial fibroblast reprogramming, ER stress, and matrix remodeling supported chiefly by cell culture and omics data (Level B). MGn cannot yet be assigned a well-supported profile; its inferences derive from comparative genomics and proteomics (Level C).

Pangenomics and machine learning are complementary hypothesis generators, not sources of mechanisms. Pangenomic analysis has produced validated results, most clearly the six MS-specific coding sequences now used as diagnostic targets. ML is most reliable for defined problems with external validation and least reliable for broad mechanistic claims built on small, heterogeneous datasets.

The principal limitation is evidentiary asymmetry. As quantified in Table 1, complete genomes number in the dozens for MG, fewer for MS, and were absent for MGn until 2026. Moreover, no published study has yet applied machine learning directly to avian *mycoplasmas*. The comparative framework is therefore only as strong as its weakest member, and any conclusion that incorporates MGn remains inferential.

Furthermore, two actions would most advance the field. The first is a complete, well-annotated MGn reference genome from a passage-controlled, phenotypically characterized strain. The second is a controlled three-species infection experiment in the same avian host, with the same protocol and analytical platforms, to replace inferences drawn from isolated single-species studies. Neither requires technology that does not yet exist; both require coordinated effort.

Medium-term priorities (including structured ML benchmarking, systematic reporting of negative validation results, and CRISPRi-based perturbation of pangenomic candidates) would build on these foundations and support an end-to-end pipeline from pangenomic target identification to controlled validation.
